# Combining α-Radioimmunotherapy and Adoptive T Cell Therapy to Potentiate Tumor Destruction

**DOI:** 10.1371/journal.pone.0130249

**Published:** 2015-06-22

**Authors:** Jérémie Ménager, Jean-Baptiste Gorin, Catherine Maurel, Lucile Drujont, Sébastien Gouard, Cédric Louvet, Michel Chérel, Alain Faivre-Chauvet, Alfred Morgenstern, Frank Bruchertseifer, François Davodeau, Joëlle Gaschet, Yannick Guilloux

**Affiliations:** 1 CRCNA—UMR 892 INSERM, Nantes, France; 2 6299 CNRS, Nantes, France; 3 Université de Nantes, Nantes, France; 4 INSERM UMR 1064 –ITUN, Nantes, France; 5 Institut de Cancérologie de l’Ouest, Saint-Herblain, France; 6 CHU Nantes, Nuclear Medicine Department, Nantes, France; 7 European Commission, Joint Research Centre, Institute for Transuranium Elements, Karlsruhe, Germany; Mie University Graduate School of Medicine, JAPAN

## Abstract

Ionizing radiation induces direct and indirect killing of cancer cells and for long has been considered as immunosuppressive. However, this concept has evolved over the past few years with the demonstration that irradiation can increase tumor immunogenicity and can actually favor the implementation of an immune response against tumor cells. Adoptive T-cell transfer (ACT) is also used to treat cancer and several studies have shown that the efficacy of this immunotherapy was enhanced when combined with radiation therapy. α-Radioimmunotherapy (α-RIT) is a type of internal radiotherapy which is currently under development to treat disseminated tumors. α-particles are indeed highly efficient to destroy small cluster of cancer cells with minimal impact on surrounding healthy tissues. We thus hypothesized that, in the setting of α-RIT, an immunotherapy like ACT, could benefit from the immune context induced by irradiation. Hence, we decided to further investigate the possibilities to promote an efficient and long-lasting anti-tumor response by combining α-RIT and ACT. To perform such study we set up a multiple myeloma murine model which express the tumor antigen CD138 and ovalbumine (OVA). Then we evaluated the therapeutic efficacy in the mice treated with α-RIT, using an anti-CD138 antibody coupled to bismuth-213, followed by an adoptive transfer of OVA-specific CD8^+^ T cells (OT-I CD8^+^ T cells). We observed a significant tumor growth control and an improved survival in the animals treated with the combined treatment. These results demonstrate the efficacy of combining α-RIT and ACT in the MM model we established.

## Introduction

Radiation therapy is one of the most efficient form of cancer therapy, and is used in the treatment of more than half of all cancer patients [1,2]. Ionizing radiation is known for its direct cytotoxic action on tumor cells [3] as well as the radiation-induced bystander effects which can destroy surrounding malignant cells [4–6]. Furthermore, impact of local radiotherapy on tumor immunity and immune cell activation has also been documented. Indeed ionizing radiation delivered on tumor cells and on the tumor cell microenvironment induce increased expression of MHC-peptide complexes [7–9], death receptor [10] as well as the release of various danger signals such as Heat shock proteins (HSPs), danger associated molecular patterns (DAMPs), or others cytokines [11,12]. Interestingly, several studies have demonstrated that radiation therapy can induce tumor regression through the development of an adaptive immune response dependent on tumor-specific T-lymphocytes [8,13–15]. These studies gave the first hints that radiation therapy and immunotherapies which had been so far envisioned as separate cancer treatment approaches could actually be combined to provide an enhanced anti-tumor response. During the last two decades, the improved understanding of cancer pathogenesis has led to the extensive development of various active and passive immunotherapy strategies. While active immunotherapies, like cancer vaccines, attempt to stimulate the patient immune system to trigger an anti-tumor response, passive immunotherapies involve the injection of molecules (e.g. antibodies) or immune cells to directly target the tumor cells [16]. Adoptive T-cell transfer (ACT) is a passive immunotherapy consisting in the infusion of large number of autologous or allogeneic lymphocytes with antitumor activity which have been amplified *ex vivo* [17]. Such approach has been largely investigated in melanoma patients through reinfusion of autologous tumor infiltrating lymphocytes (TIL) [18]. Also ACT on its own can induce an anti-tumor response, several preclinical and clinical studies have demonstrated that its efficiency was strenghtened when combined with external irradiation [19–22]. Besides inducing lymphodepletion, ionizing radiation was shown to enhance ACT efficacy by raising tumor immunogenicity and by promoting an abscopal effect which consists in the inhibition of distant tumors after local irradiation [8,20,22].

Radioimmunotherapy (RIT) represents a selective internal radiation therapy suited for the treatment of disseminated cancers. RIT involves the use of a monoclonal antibody (mAb) to deliver radionuclides directly to the targeted tumor cells [23–26]. In the clinic, this approach has been particulary successful for the treatment of non-Hodgkin lymphoma patients using anti-CD20 mAb coupled to two β-emitters, Ytrium-90 (Zevalin) and Iodine-131 (Bexxar) [27]. α-particles which are highly cytotoxic agents are also under development for clinical applications. α-emitters are characterized by a high linear energy transfer (LET), in the range of 100 KeV/μm, a very high energy deposit over a very short path length (50–100 μm) and are poorly sensitive to hypoxia [28]. Therefore, such type of radionuclide is very efficient for targeting and killing disseminated tumor cells with minimal impact on healthy tissues and can be used in the treatment of different type of cancers [29,30] and in particular in multiple myeloma (MM) [31]. To date, *in vivo* interactions of α-radiation with the immune system, such as the release of DAMPs, for example, have been sparsely documented. We recently demonstrated that bismuth-213, an α-emitter, can induce immunogenic cell death of cancer cells and stimulate an anti-tumor response [32]. Furthermore, very few studies combining α-RIT and immunotherapy have been performed [33]. Hence, we sought to investigate combining α-RIT and ACT, to study if, in this specific setting, irradiation could also reinforce the immunotherapy approach and help promote an efficient and long-lasting anti-tumor response.

In order to perform this study, we needed a tumor model expressing both a target antigen for α-RIT and a specific MHC-peptide complex that can be recognized by tumor-specific T-cells. Therefore, we established an immunocompetent MM mouse model, where the tumor is induced by injection of the 5T33 MM cell line, which expresses the CD138 antigen (syndecan-1), a hallmark of all MM cells, and ovalbumin (OVA). α-RIT was then performed by injection of an anti-CD138 antibody coupled to bismuth-213. Subsequently, ACT consisted in the injection of OVA-specific CD8^+^ T cells (OT-I CD8^+^ T cells). Here, we report that this model is suitable to study α-RIT and ACT combination, and that OT-I CD8^+^ T-cells migrate to the tumor site. In correlation, with those data, we demonstrated that such combined treatment results in a significant tumor growth inhibition associated with an improved survival.

## Materials and Methods

### Animals

Female C57BL6/KaLwRij mice (Harlan CPB, Horst, The Netherlands) were used for tumor engraftment and treatments. Animals were housed in UTE animal facility (SFR François Bonamy, IRS-UN, University of Nantes, licence number: B-44-278), under conventional conditions. All mice were 8 to 10 weeks old at the beginning of each experiment. Experiments performed in this study were approved by the Animal Experimentation Ethic Comittee of the Pays-de-Loire (protocol n° CEEA.2013.2).

OT-I transgenic mice were used to get the OT-I CD8^+^ T cells for ACT. These mice were obtained by first intercrossing OT-I TCR-transgenic mice (C57BL/6-Tg(TcraTcrb)1100Mjb/Crl) (Charles River, France) and CD45.1 congenic mice (B6.SJL-Ptprca Pepcb/BoyCrl) (Charles River, France) in order to track OT-I CD8^+^ T cells in vivo using the CD45.1 expression. These mice were subsequently bred to C57BL/6 Rag1KO mice (CERFE—GIP Genopole, France, obtained through A. Savina, Curie Institute, France) and further intercrossed to generate an OT-I.CD45.1Rag1KO mouse line homozygous for each allele. Animals were housed and intercrossed at the INSERM UMR 1064—ITUN specific pathogen free animal facility.

### 5T33-OVA Multiple Myeloma Cells

5T33-OVA cell line (Ministère de l'enseignement supérieur et de la recherche, agreement n° 5663) was produced by modification of the original 5T33 murine MM cell line [34,35] kindly provided by Dr. Harvey Turner (Department of Nuclear Medicine, Fremantle Hospital, Western Australia) with the permission of Dr. J. Radl (TNO Institute, Leiden, The Netherlands). A sequence containing the cDNA coding for cytoplasmic Ovalbumin was obtained by BamHI-EcoRI digestion of the cytoplasmic Ovalbumin vector (Addgene). The fragment was cloned into BamHI and EcoRI sites of the retroviral vector pMX [36]. Phoenix-Ampho packaging cells (ATCC® CRL-3213) were then transiently transfected with the ovalbumin retroviral construct, supernatants were collected and used to transduced 5T33 cells. Three days after retroviral transduction, 5T33-OVA cells were sorted by flow cytometry (FACS ARIA III, BD) by using mAb 25-D1.16 (eBioscience), specific for H_2_K^b^ /OVA_257–264_ (SIINFEKL) complexes.

5T33-OVA cells were cultured in RPMI1640 medium (Gibco) containing 2 mM L-glutamine, and 10% heat-inactivated fetal calf serum (PAA) and were incubated at 37°C, 5% CO2, 95% humidity. Aliquots of early passaged cells were frozen in 10% dimethylsulfoxide, 90% FCS and stored under liquid nitrogen.

### 5T33-OVA Multiple Myeloma model

Syngeneic C57BL6/KaLwRij mice were subcutaneously injected with 2x10^6^ 5T33 or 5T33-OVA in the right flank. On day 10 after tumor implantation, mice received α-RIT treatment and on day 11 after tumor implantation, mice received ACT by intravenous injection (i.v.) of OT-I CD8^+^ T cells into the tail. Health status of tumor-bearing mice was monitored daily, and tumor growth was measured at specific time points using caliper. The mice were sacrificed when tumour reached a volume of 2500 mm^3^.

### OT-I cell generation and adoptive cell transfer (ACT)

Spleen and lymph nodes from OT-I transgenic mice were extracted under aseptic conditions and dissociated into single-cell suspensions. OT-I CD8^+^ T cells were subsequently purified using CD8a^+^ T Cell Isolation Kit II (Miltenyi Biotech, Bergish Gladbach, Germany) according to the manufacturer instructions. Efficacy of the selection was controlled by flow cytometry; the resulting cell suspension typically contained more than 80% of OT-I CD8^+^ T cells.

Stimulation of OT-I CD8^+^ T cells was done by incubating 1x10^6^ purified OT-I CD8^+^ T cells with 5x10^6^ irradiated syngeneic C57BL6/KaLwRij mice spleen cells in 2 ml DMEM supplemented with 10% FCS (Life Technologies, Paisley, Scotland) containing 5μM Ovalbumin peptide SIINFEKL (OVA_257–264_), 5U/mL IL-2, and 20ng/ml murine IL-12. On day 3, the cultures were split into four aliquots and fed with fresh medium containing 5U/mL IL-2. On day 6, cells were harvested and washed with DMEM medium at least three times. Eleven days after 5T33-OVA MM cells graft, activated OT-I CD8^+^ T cells were adoptively transferred into mice.

### Cytotoxicity assay

Before ACT, OT-I CD8^+^ T cell effector function was assessed using a standard ^51^Cr-release assay,against different target cells: 5T33, 5T33-OVA and 5T33 cells pulsed with 1μM OVA_257–264_ peptide. Target cells were labeled with 75 μCi ^51^Cr for 1 h at 37°C, washed four times with culture medium, and then plated at the indicated effector to target (E:T) cell ratio in a 96-well V-bottom plate. After a 4-h incubation at 37°C, 25 μl of supernatant were removed from each well, mixed with 100 μl of scintillation fluid, and ^51^Cr activity was counted in a scintillation counter (MicroBeta, Perkin Elmer). Each test was performed in triplicate. The results are expressed as the percentage of lysis, which is calculated according to the following equation: (experimental release − spontaneous release)/(maximal release − spontaneous release) × 100, where experimental release represents the mean cpm for the target cells in the presence of effector cells, spontaneous release represents the mean cpm for target cells incubated without effector cells, and maximal release represents the mean cpm for target cells lysed with 1% Triton X-100 (Sigma).

### Antibody Radiolabeling and α-RIT treatment

The rat anti-mouse CD138 antibody was conjugated to 2-(4-isothiocyanatobenzyl)-cyclohexyl-diethylenetriaminepenta-acetic acid (SCN-CHX-A”-DTPA, Macrocyclics, Dallas, TX, USA). For radiolabeling, 100 μg of this immunoconjugate was incubated with 160 ± 6 MBq ^213^Bi eluted from a ^225^Ac/^213^Bi generator (Institute for Transuranium Elements, Karlsruhe, Germany) for 10 min at 37°C in 0.6 M sodium acetate (pH 5.3) and 0.01% ascorbic acid. The resulting ^213^Bi-labeled immunoconjugate was purified from unbound ^213^Bi by size exclusion chromatography using a PD-10 column (Sephadex G-25). Radiochemical purity was >95%, as determined by ITLC-SG using 0.1M citrate buffer pH 4.5. Treatment was initiated 10 days after tumor engraftment by i.v. injection in the tail vein of 3.7 MBq ^213^Bi-labeled anti-mouse CD138 antibody (specific activity 946 ± 35 kBq/μg).

### Antibodies and flow cytometry

When tumor reached a volume of 2500 mm^3^, the mice were sacrificed. Tumor, lymph nodes, spleen and blood were then harvested and made into single cell suspensions by mechanical disassociation.

Fluorochrome-conjugated mouse specific mAbs directed against CD45.1, CD8b antigens, isotype control mAbs and Fc block were purchased from BD Biosciences (Le Pont de Claix, France). Fluorochrome-conjugated mAbs directed against H_2_K^b^/OVA_257–264_ complexes were purchased from eBioscience (Paris, France).

Cell surface staining was done using standard procedure in the presence of 0.1% BSA. Adequate isotypic controls were used in parallel. Stained samples were analyzed on a FacsCanto flow cytometer using Diva software (BD Biosciences) and on FacsCalibur flow cytometer using Cell Quest Pro software (BD biosciences).

### Statistical analysis

Tumor volumes were calculated via three measurements of tumor with caliper and using the formula 4/3π x r^3^. Data were represented as mean ± SD or SEM as indicated in each experiment. Comparisons between the groups were made using two-way analysis of variance (ANOVA) and by student’s t test. The median survival of mice with the different treatments was calculated by using the Kaplan–Meier method and analyzed using log rank test. A p value of <0.05 was considered significant for all the experiments.

## Results

### 5T33-OVA phenotypic analysis

**Fig 1:**
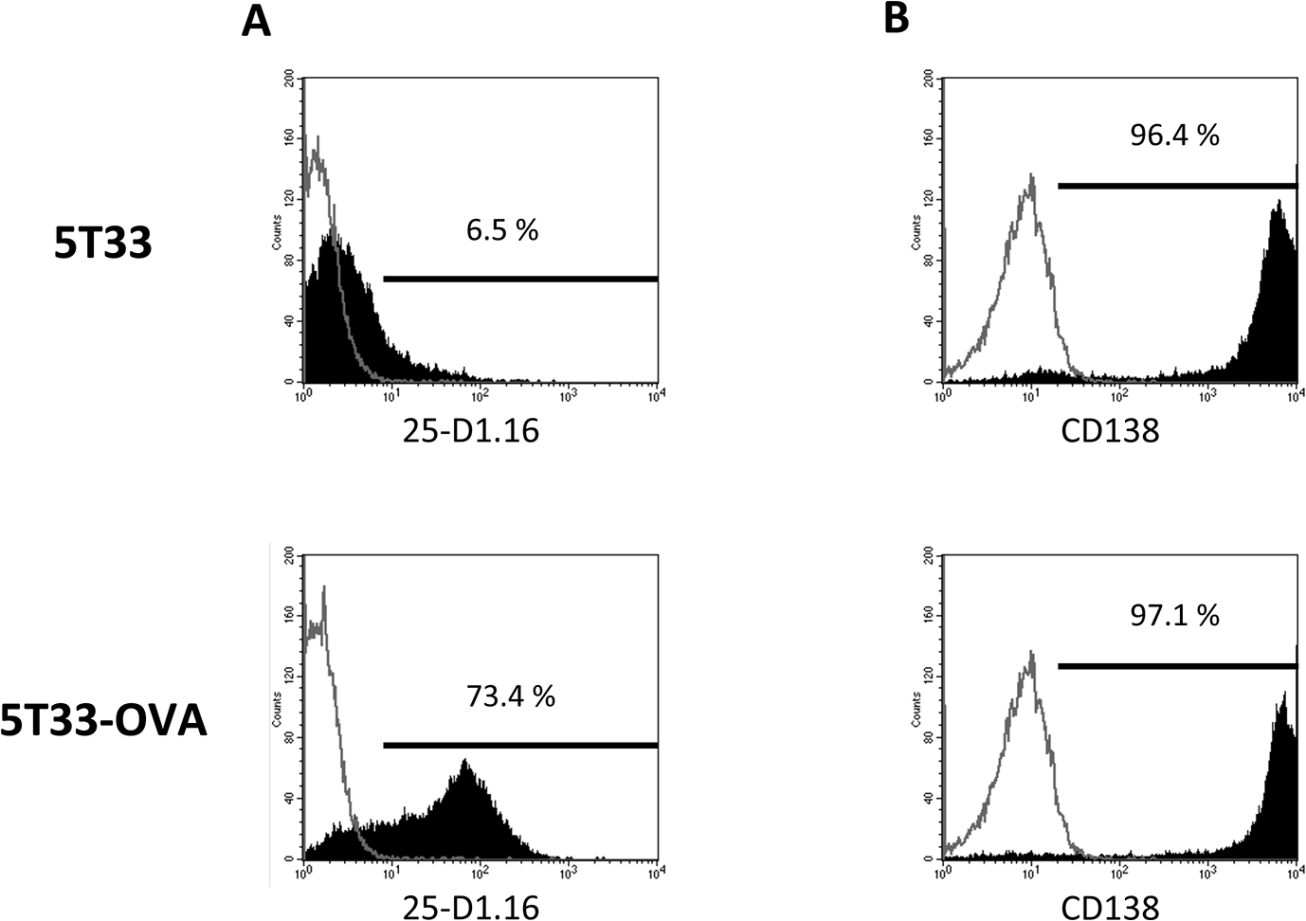
5T33-OVA phenotypic analysis after transduction by a lentiviral vector encoding cytoplasmic *ovalbumin* (A) 5T33 and 5T33-OVA staining with PE-conjugated antibody 25-D1.16, which specifically recognizes the OVA peptide ‘SIINFEKL’ bound to the MHC class I molecule H-2K^b^ and (B) staining of 5T33 and 5T33-OVA with APC-conjugated anti-mouse CD138 mAb. Flow cytometry was performed on a BD FACSCalibur Flow Cytometry System.

The prerequisite to evaluate the therapeutic effect of α -RIT and ACT combination was to develop a suitable tumor model expressing two antigens, the first one allowing targeting with a radiolabeled antibody (CD138) and the second one for specific OT-I CD8^+^ T cell recognition (H_2_K^b^/OVA_257–264_ complexes). To do so, we transduced the 5T33 cells with the cytoplasmic ovalbumin gene and the resulting 5T33-OVA cells were purified by FACS sorting based on the expression of H_2_K^b^/OVA_257–264_ complexes on the plasma membrane. The purity of the sorting was controlled by cell labeling with the 25-D1.16 antibody specific for H_2_K^b^/OVA_257–264_ complexes. As shown in Fig 1A, 73.4% of 5T33-OVA cells expressed this MHC-peptide complex compared to parental 5T33 cells. Endogeneous CD138 expression was also controlled to ensure that the transduction did not modify its expression on the 5T33-OVA cells. As shown in Fig 1B, respectively 96.4% and 97.1% of 5T33 and 5T33-OVA cells respectively were labeled by the anti-CD138 antibody. Together, these data demonstrate that 5T33-OVA cells express H_2_K^b^/OVA_257–264_ complexes without altering the expression of CD138 antigen at the cell surface.

### *In vitro* Recognition of 5T33-OVA cells

**Fig 2:**
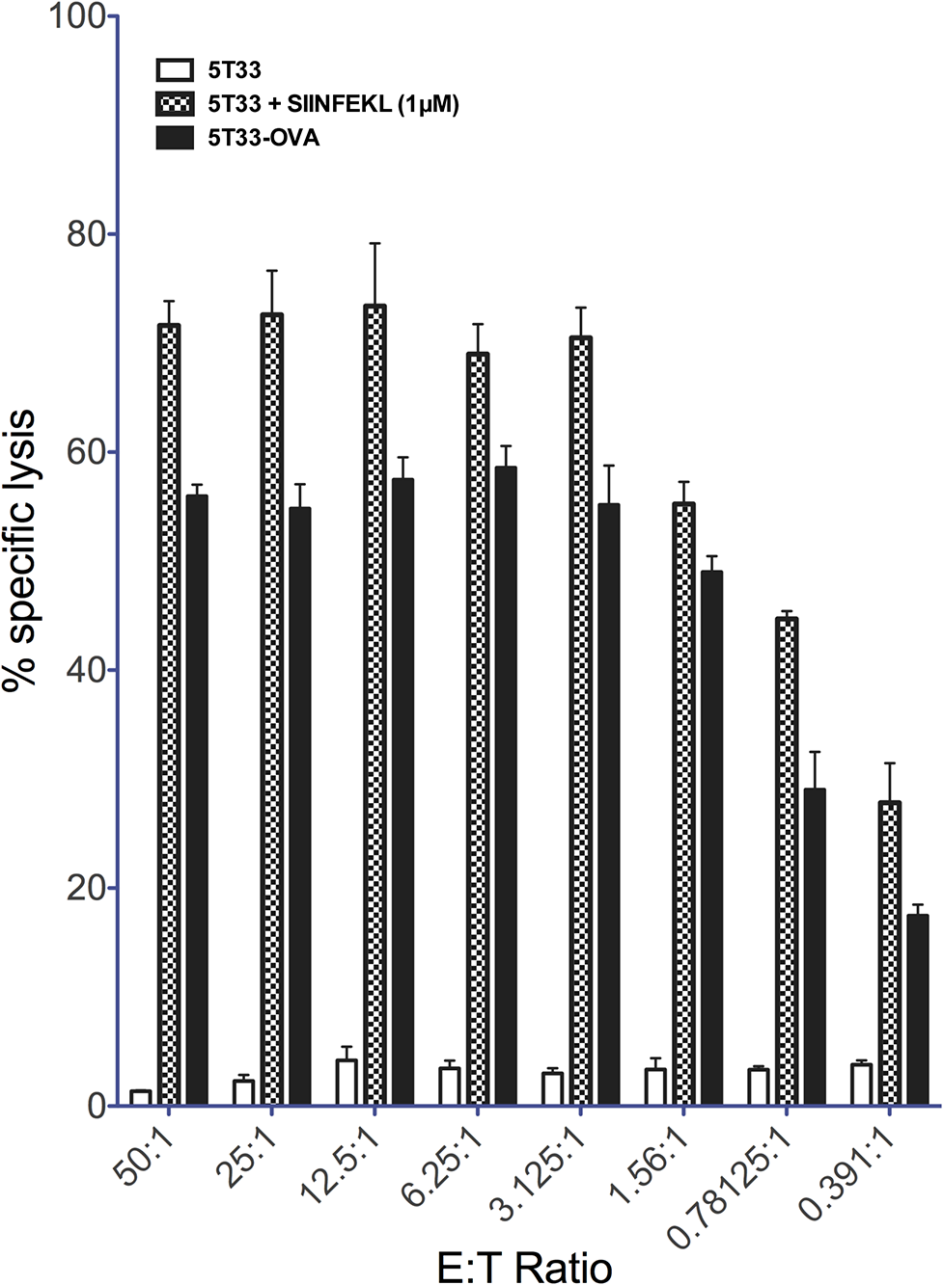
*In vitro* recognition of 5T33-OVA. Cytotoxic T-cell-mediated lysis of 5T33-OVA targets. T cells isolated from OT-I mice were activated using irradiated splenocytes loading with OVA_257–264_ (SIINFEKL) peptide. *In vitro* T-cell-mediated cytotoxicity against 5T33-OVA was determined using a standard four-hour ^51^Cr release assay at several effector-to-target ratios (E:T Ratio).

To further characterize the 5T33-OVA cells, we performed a cytotoxicity assay to evaluate the recognition efficiency by OT-I CD8^+^ T cells. For this experiment, we primed OT-I CD8^+^ T-cells *in vitro* for 5 days. As shown in Fig 2, primed OT-I CD8^+^ lymphocytes did not kill parental 5T33 cells (<5%), whereas 5T33-OVA cells and 5T33 pulsed with OVA_257–264_ peptide were killed, with lysis of 17% to 59% and 28% to 73% respectively at the different E:T ratios. These data indicate that the 5T33-OVA express enough H_2_K^b^/OVA_257–264_ complexes to be recognized by the OT-I T-cells. Moreover, we observe an efficient recognition of 5T33-OVA cells by OT-I T-cells of 5T33-OVA cells in association with the high level of H_2_K^b^/OVA_257–264_ expression on 5T33-OVA cells seen by flow cytometry (Fig 1A).

### 5T33-OVA *in vivo* tumor model

**Fig 3:**
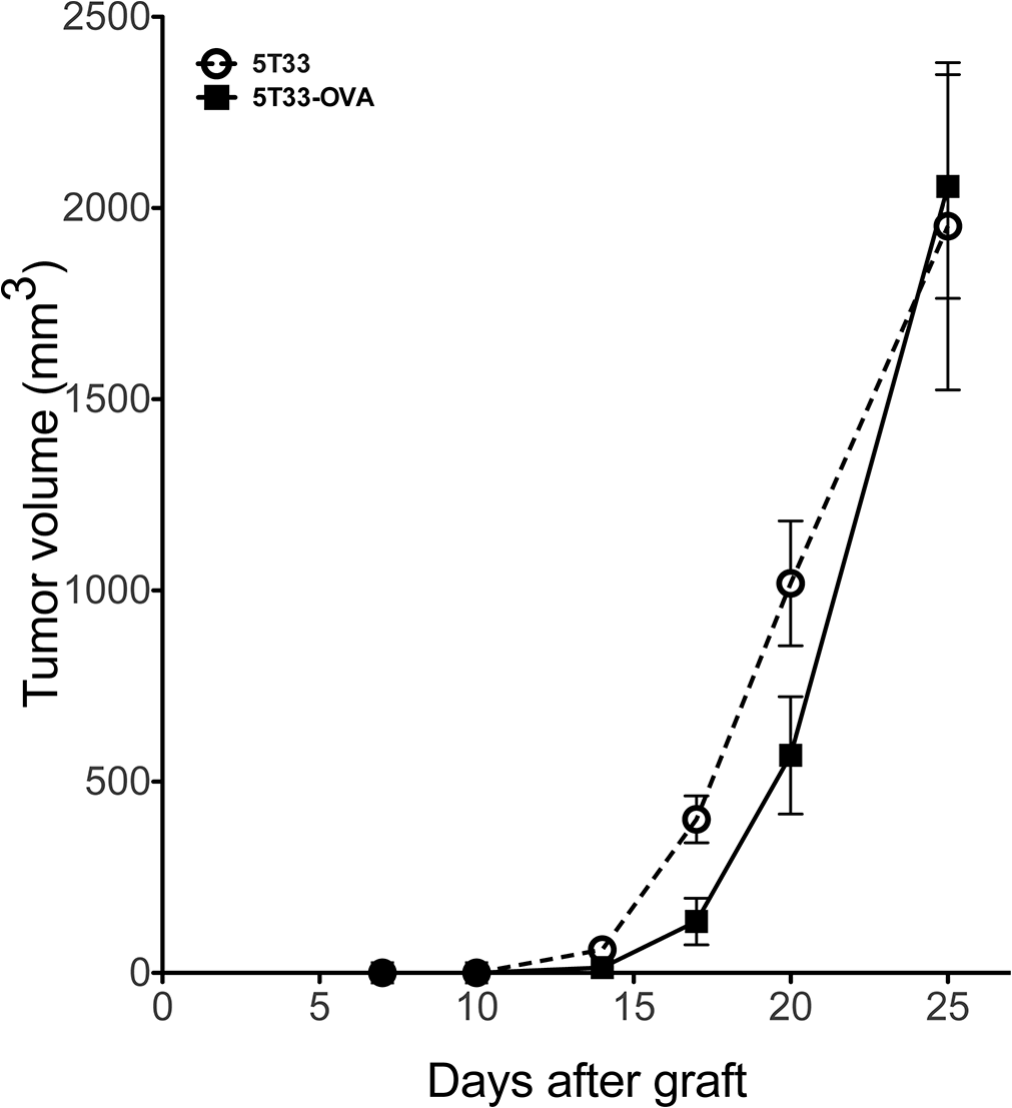
*In vivo* tumor growth of 5T33-OVA. Growth comparison of established tumors 5T33 versus 5T33-OVA. Animals were injected subcutaneously with 2x10^6^ tumor cells (n = 5 mice per group).

To complete the validation of our model, we investigated the *in vivo* growth capability of 5T33-OVA after subcutaneous injection. Such study was performed by injection of 2x10^6^ of 5T33 or 5T33-OVA in the right flank of C57BL6/KalwRij mice and tumor progression was monitored. As shown in Fig 3, no growth difference was detected between parental 5T33 and 5T33–OVA (2 way ANOVA, p<0,05). These data show that there is a minimum impact of OVA expression on tumor cell development *in vivo*.

### ACT using OT-I

**Fig 4:**
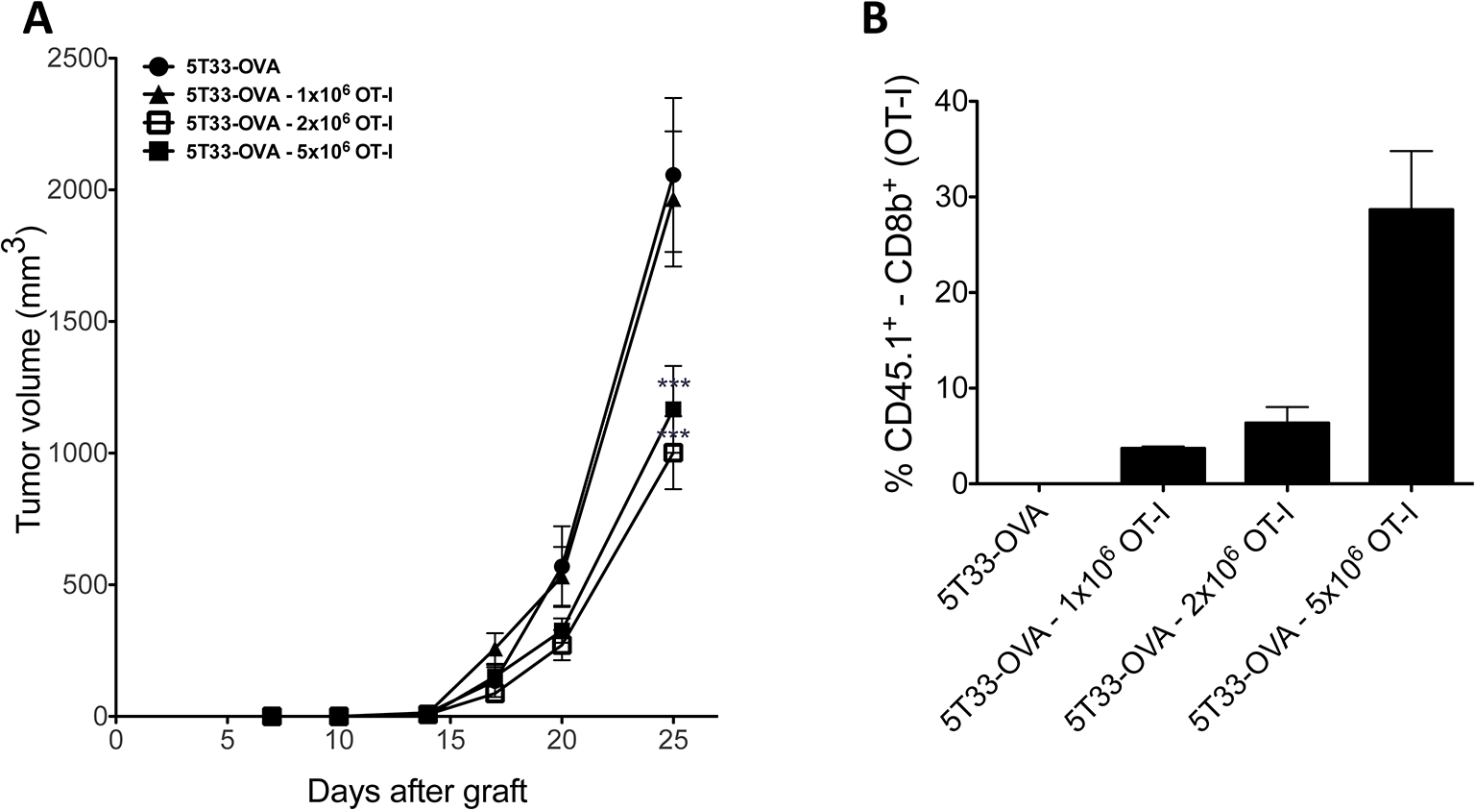
Adoptive OT-I T cell transfer. (A), Dose response of 5T33-OVA tumor cells to adoptive transfer of OT-I CD8+ T cells treatment. Animals were injected subcutaneously with 2x10^6^ tumor cells and received the indicated doses of OT-I CD8^+^ T cells (n = 10 mice per group). Tumor volume was determined by using a caliper. Data points represent mean ± SD of 10 measures. *** p<0,001 as determined by two-way ANOVA and Bonferonni post-tests. (B), Tumors were explanted and single cell suspensions were prepared by grinding tumors in a tissu grinder. Cells were stained with monoclonal anti-CD8b PE and anti-CD45.1 FITC. Histograms represent the percentage of CD45.1^+^ in CD8^+^ cells.

To assess the impact of ACT on tumor growth, 1x10^6^, 2x10^6^ or 5x10^6^ OT-I CD8^+^ T cells were injected intravenously in the mice tail vein, 11 days after subcutaneous tumor engraftment. As shown in Fig 4A, at day 25, mice injected with 2 or 5x10^6^ OT-I CD8^+^ T cells exhibited a significant tumor growth reduction compared with mice receiving no treatment or receiving 1x10^6^ OT-I CD8^+^ T cells (p< 0.001). There was no significant difference between untreated mice and those receiving 1x10^6^ OT-I CD8^+^ T cells (p>0.05).

OT-I CD8^+^ T cells express the CD45.1 congenic marker while the C57BL6/KaLwRij mice are homozygous for the CD45.2 alloantigen. Thus the CD45.1 antigen enabled us to evaluate the ability of OT-I CD8^+^ T cells to infiltrate the tumor *in vivo*. T-cell infiltrates were analyzed when the mice were sacrificed in between 25 and 27 days after tumor engraftment (Fig 4B). Tumors were mechanically dilacerated and cell suspensions were analyzed by flow cytometry to quantify OT-I CD8^+^ T cells. The injected OT-I CD8^+^ T cells were detectable in the tumor up to 14 days after injection and the proportion of OT-I CD8^+^ T cells among CD8^+^ T cells was higher in mice injected with 5x10^6^ OT-I CD8+ T cells compared to mice injected with 1x10^6^ or 2x10^6^ OT-I CD8^+^ T cells.

These results demonstrated the ability of injected OT-I CD8+ T cells to reach and infiltrate the tumor. Since the injection of 2 or 5x10^6^ OT-I CD8+ T cells had similar effects on tumor progression without toxicity, we decided to perform the ACT with 5x10^6^ OT-I CD8+ T cells in order to optimize therapeutic efficacy.

### Combining α-RIT + ACT is the most efficient treatment towards tumor development

Next, we investigated whether the combination of α-RIT with ACT could efficiently inhibit tumor growth and increase survival of mice grafted with 5T33-OVA. α-RIT was performed 10 days after tumor engraftment at a time where 60.5% of the animals (23 mice on 35) had a small established tumor, with a mean volume of 17,67 ± 5,26 mm^3^. Since the radionuclide half-life is short (45.6 minutes), ACT was done 1 day after RIT, i.e 11 days after 5T33-OVA engraftment. Tumor growth was also evaluated for each treatment alone.

**Fig 5:**
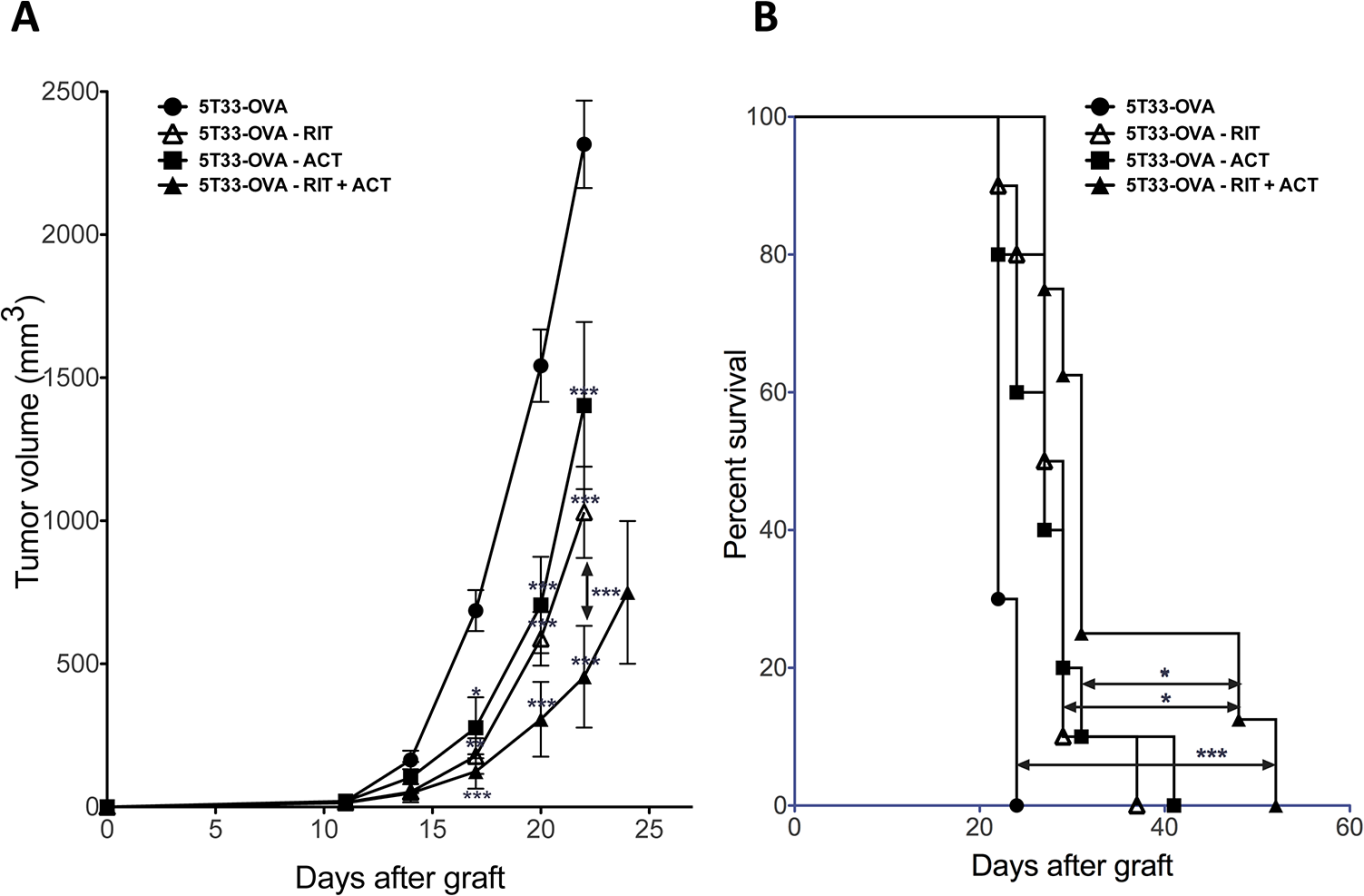
Combination of RIT with adoptive immunotherapy inhibits tumor growth and prolongs survival. (A), Animals were injected with 2x10^6^ tumor cells subcutaneously and received the indicated treatment (n = 8 to 10 mice per group). Tumor volume was measured. Data points represent mean ± SD. * p<0,05, ** p<0,01, *** p<0,001 as determined by two-way ANOVA and Bonferonni post-tests. The combination of RIT + ACT or RIT alone or the ACT transfer alone significantly decrease tumor growth when compared with the control. The combination of RIT + ACT significantly decrease tumor growth when compared RIT alone or the ACT transfer alone. (B), Animals were injected subcutaneously with 2x10^6^ tumor cells and received the indicated treatment. The percentage of surviving mice was evaluated when tumor volume reach end-point of 2500 mm^3^. The combination of RIT + ACT significantly increased survival (median survival of 31 days; log-rank, 0.0001) when compared with control or RIT alone (median survival of 28 days; log-rank, 0,0413) or the ACT transfer alone (median survival of 27 days; log-rank, 0,0391) cohorts. Statistical analysis were performed using non-parametric Mann-Whitney test.

No significant difference was observed between tumor growth after α-RIT treatment alone or ACT treatment alone (Fig 5A). However, mice receiving the combination of α-RIT followed by ACT exhibited a significant decrease in tumor development compared to the control mice that received PBS or the ones that received RIT alone or ACT alone. Monitoring of those animals was also performed with regards to their survival after each treatment; the mice being sacrificed when tumour reached an endpoint volume of 2500 mm^3^ (Fig 5B). Once again, there was a significant difference in median survival of mice received the combination of RIT and ACT (31 days) compared to mice receiving ACT alone (28 days, p = 0.0391), or RIT alone (27 days, p = 0.0413). Altogether these data demonstrated that combining RIT and ACT was the most efficient therapeutic approach in terms of tumor development and survival. We investigated the impact of irradiation with α -particles on MHC class I or H_2_K^b^/OVA_257–264_ complex expresssion on 5T33-OVA cells *in vitro* (S1 File). No clear variation in the overall MHC class I expression was observed (Figure A in S1 File), however irradiation resulted in a transient increase of the H_2_K^d^/OVA_257–264_ complex expression (Figure B in S1 File) that is specifically recognized by OT-I CD8+ T cells and which might contribute to therapeutic efficacy of the combined treatment.

### OT-I CD8+ T cells persist within the tumor but also in the lymphoid organs and the periphery

**Fig 6:**
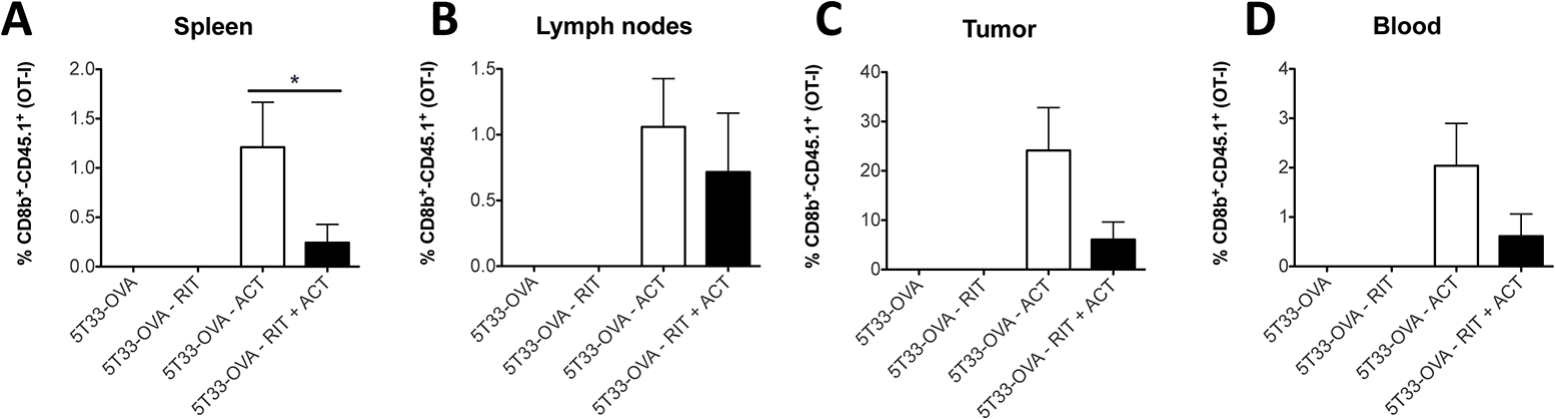
OT-I CD8^+^ T cells persist within the tumor but also in the lymphoid organs and the periphery. After the different treatment as indicated on the graph (n = 8 to 10 per group), when tumour reached a volume of 2500 mm^3^, the mice were sacrificed and spleen (A), lymph nodes (B), tumor (C) and blood (D) were harvested. Then, cells were stained with monoclonal anti-CD8b PE and anti-CD45.1 FITC. Histograms represent the percentage of CD45.1^+^ in CD8^+^ cells. Statistical analysis were performed using non-parametric Mann-Whitney test.

To investigate the role of OT-I CD8+ T cells on tumor growth *in vivo*, we analyzed their persistence in the tumor as well as in the spleen, lymph nodes and in the blood, at the end point of the experiment (when the tumor reached 2500 mm^3^). Following treatment with ACT alone, OT-I CD8+ T cells represented 24% of CD8^+^ cells in the tumor compare to only 6% when ACT was combined with α-RIT (Fig 6). Surprisingly, the therapeutic efficacy in this experiment was inversely correlated with the amount of OT-I CD8+ T cells found in the different organ we analyzed. Indeed, the anti-tumor response was most effective after α-RIT combined with ACT than after ACT alone. This suggest that OT-I CD8+ T cells disappear faster when the therapeutic efficacy is higher.

## Discussion

RIT and immunotherapy are two types of strategies developed for the treatment of diverse solid and disseminated cancers [29]. On one hand, RIT has been evaluated in animal models as well as in humans and has emerged as one of the most promising treatment option, particularly for hematologic malignancies [37]. However, despite progress in targeting and vectorisation, new combined therapies need to be evaluated in order to improve disease outcome. On the other hand, although many immunotherapies (e.g cytokines, cell therapies, tumor vaccines…) show potent anti-tumor responses *in vitro*, malignant cells can evade the immune system and produce factors that attenuate anti-tumor effects elicited by those immunotherapies [16].

Tumor response to vectorized or non vectorized radiation can lead to the emergence of an immune response including T lymphocyte-mediated immune response [15,38]. Moreover, radiation has a general impact on the tumor cells and immune system: enhancing peptide production, the expression of Fas [10], ICAM-I [7] and as well as MHC class I [8] and tumor antigen [7] expression in the tumor. These events result in a better recognition of the tumor by CTL.

Tumor-specific antibodies coupled to α-particles can mediate tumor destruction by delivering radiation on the tumor site with acceptable exposure to surrounding healthy tissues and lead to increased tumor immunogenicity [32,33]. This prompted us to combine α-RIT with CD8^+^ T cells targeting a specific MHC class I peptide complex expressed on the tumor, also called ACT. This novel combination may potentiate α-RIT and add a new perspective to established cancer treatments.

Thus, this study aimed to develop a tumor model in order to evaluate the relevance of combining α-RIT and ACT consisting in the injection of tumor-specific T lymphocytes. This therapeutic association was evaluated in the immunocompetent C57BL6/KaLwRij with 5T33-OVA MM mouse model. Although orthotopic development of the disease is possible in this model, we chose to study subcutaneous MM grafts, a setting that facilitates the tumor growth evaluation and also the monitoring of transferred T cells, especially lymphocytes inside the tumor. Injection of OT-I CD8^+^ T cells was performed 24 hours after α-RIT to benefit from the early effects of ionizing radiation on the tumor (e.g inflammatory context, release of cytokines and/or DAMPs…) without any damage to the transferred T cells because bismuth half-life is very short. The high-linear energy transfer (LET) characteristics of α -particles (50 to 230 kEV/μm) allows very localized irradiation and efficient tumor cell toxicity [39]. However the short path length (50 to 100μm) of these particles makes them more suitable for disseminated or tiny clusters of tumor cells [40]. Therefore it is important to notice that in this study, the therapeutic interventions were conducted 10 and/or 11 days after MM engraftment at a time where tumor were already established in the majority of the animals (mean volume = 17,67 ± 5,26 mm^3^). Despite this sub-optimal setting, α-RIT alone or in combination with ACT was significantly efficient to delay tumor growth.

The results show that OT-I CD8^+^ T cells persist over time, that they have the capabilities to migrate within the tumor the tumor and that their tropism for the tumor is dose-dependent (Fig 4B). Persistence of effector T cells *in vivo* is an essential parameter in cancer treatment because it can lead to enhanced antitumor efficacy *in vivo* [41,42]. Therefore to better understand the implication of the OT-I CD8^+^ T cells in the anti-tumor response, we analyzed their persistence in different tissues of the animals at the end point. Interestingly and even though the combined treatment provides the best therapeutic efficacy, we observed less OT-I CD8^+^ T cells in the spleen, lymph nodes, tumors and peripheral blood of mice treated with α-RIT combined with ACT than in the one that received ACT alone. Several hypotheses might explain such result. First, for each mouse this study was performed at the end point, and the mice receiving with the combined treatment were sacrified later than the others. During these few additionnal days of survival, OT-I CD8^+^ T cells could have died progressively resulting in a lower amount at the time of analysis. On the other hand, because of the α-RIT and the inflammatory and/or immunological (e.g. Figure B in S1 File and the slight increase of H_2_K^b^/OVA_257–264_ complex expression we observed on 5T33-OVA cells after irradiation) context induced around the tumor, the activated OT-I CD8^+^ T cells might exert their anti-tumor activity in a larger extent and more efficiently than after ACT alone. As a result the OT-I CD8^+^ T cells could be more sensitive to activation induced cell death (AICD) [43]. Finally, this data could also reflect the exhaustion of OT-I CD8^+^ T cell effector functions induced by the tumor immunosuppressive environment (e.g. expression of PDL-1, Fas-L, immunosuppressive cytokines, Treg cells). In mice treated with ACT alone, OT-I CD8^+^ T cells would then migrate and accumulate in the different tissus without destroying the tumor cells.

OT-I CD8^+^ T cells have been stimulated the same way *in vitro* for both ACT alone or ACT combined with RIT. Only the therapeutic combination resulted in a better tumor growth control. These results support the hypothesis that α-RIT can induce an inflammatory context in the tumor and its microenvironnement [32] that favor infiltration and function of the injected-OT-I CD8^+^ T cells.

This inflammatory context could potentiate the transition of activated T cells to optimal induction of effector T cells and thus facilitate tumor growth control shortly after α-RIT. Such hypothesis has already been proposed in a mastocytoma tumor model [44]. In addition, a transient hematologic toxicity is observed a few days after α-RIT (nadir = between 6 and 15 days after α-RIT) as a result of bismuth irradiation on blood cells and bone marrow [31]. This peripheral blood cell depletion, especially the lymphodepletion could therefore promote OT-I T cell expansion and activation. Finally, it is also possible that radiation-induced remodeling of the abnormal tumor vessels results in efficient tumor infiltration by adoptive T cells and a better control of tumor proliferation [45].

In summary, the current study explored the feasibility, safety and efficacy of combining ACT after α-RIT, demonstrating a significant better tumor growth control and improved survival compared to α-RIT or ACT alone. Moreover this combination showed no adverse effects, especially no additional toxicity as demonstrated by the absence of early mortality in mice receiving ACT after α-RIT.

## Supporting Information

S1 File Expression of MHC class I and H_2_K^b^/OVA_257–264_ complexes on 5T33-OVA cells after irradiation with -particles. The ^213^Bi labeled 9E7.4 mAb was added to 5T33-OVA cells in culture medium at a final activity of 44.4, 103.6 or 266.4 kBq/mL for *in vitro* studies. Then cells were harvested at 24, 48 and 72 hours after irradiation. Cells were stained by biotin-conjugated mAb against H_2_K^b^/H_2_D^b^ (BD Biosciences, Le Pont de Claix, France) and revealed with streptavidin-PE (BD Biosciences, Le Pont de Claix, France) or by Fluorochrome-conjugated mAb directed against H_2_K^b^/OVA_257–264_ complexes (eBioscience Paris, France). After staining, cells were fixed in paraformaldehyde 1%. Cell surface staining was done using standard procedure in the presence of 0.1% BSA. Adequate isotypic controls were used in parallel. Stained samples were analyzed on FacsCalibur flow cytometer using Cell Quest Pro software (BD biosciences). Analysis of RFI for (**Figure A in S1 File**) mouse MHC-I (H_2_K^b^–H_2_D^b^) and (**Figure B in S1 File**) MHC-OVA complex (H_2_K^b^/OVA_257–264_). RFI is calculated as mean of fluorescence intensity of the specific antibody divided by that of negative cells.
(TIF)
