# Supplementary figures and images for: Combining α-Radioimmunotherapy and Adoptive T Cell Therapy to Potentiate Tumor Destruction
Source: PLoS One. 2015 Jun 22;10(6):e0130249. doi: 10.1371/journal.pone.0130249 (PMC4476754; doi:10.1371/journal.pone.0130249)

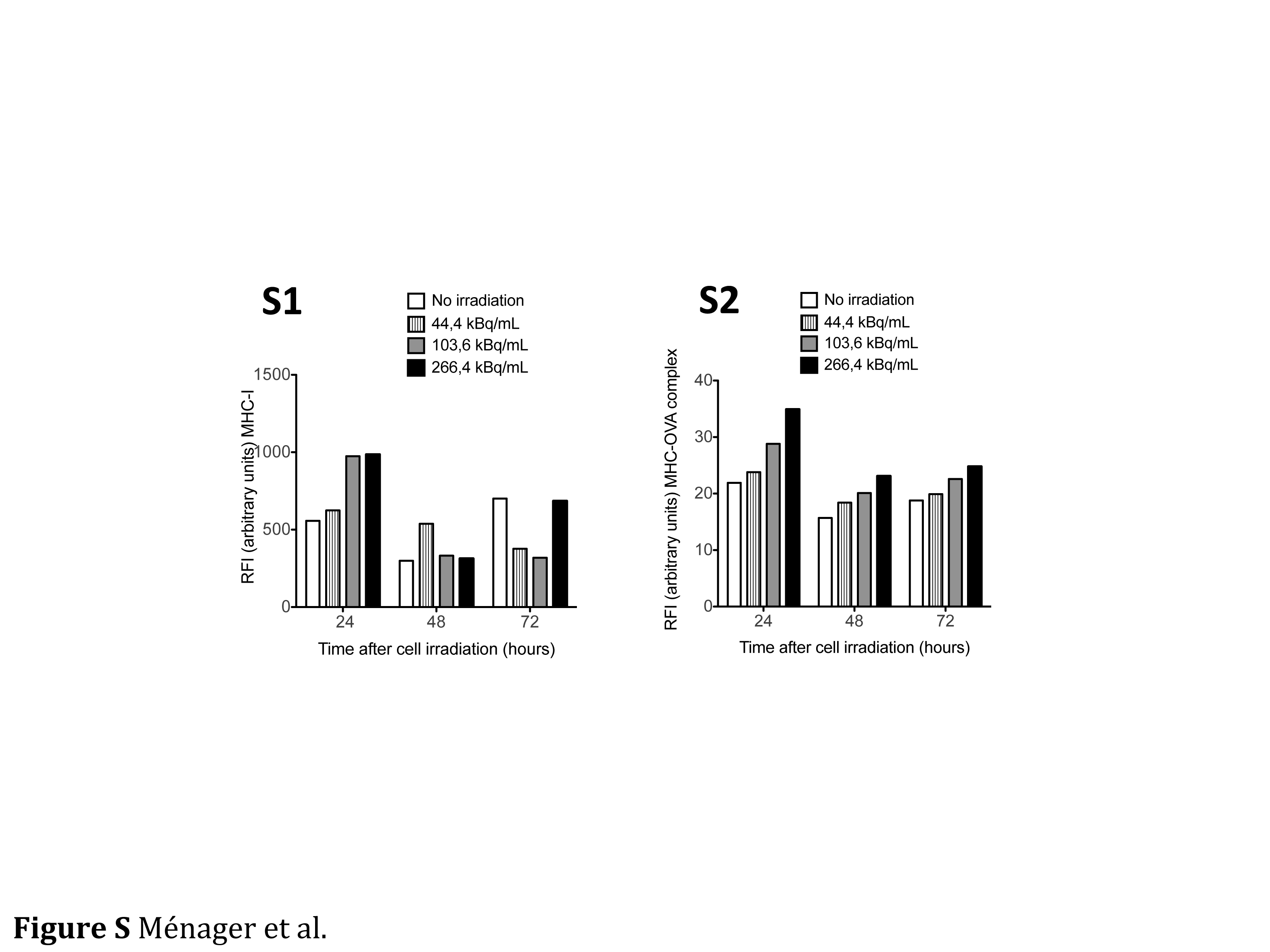

Supplement: S1 File — The 213Bi labeled 9E7.4 mAb was added to 5T33-OVA cells in culture medium at a final activity of 44.4, 103.6 or 266.4 kBq/mL for in vitro studies. Then cells were harvested at 24, 48 and 72 hours after irradiation. Cells were stained by biotin-conjugated mAb against H2Kb/H2Db (BD Biosciences, Le Pont de Claix, France) and revealed with streptavidin-PE (BD Biosciences, Le Pont de Claix, France) or by Fluorochrome-conjugated mAb directed against H2Kb/OVA257–264 complexes (eBioscience Paris, France). After staining, cells were fixed in paraformaldehyde 1%. Cell surface staining was done using standard procedure in the presence of 0.1% BSA. Adequate isotypic controls were used in parallel. Stained samples were analyzed on FacsCalibur flow cytometer using Cell Quest Pro software (BD biosciences). Analysis of RFI for (Figure A in S1 File) mouse MHC-I (H2Kb–H2Db) and (Figure B in S1 File) MHC-OVA complex (H2Kb/OVA257–264). RFI is calculated as mean of fluorescence intensity of the specific antibody divided by that of negative cells. (TIF) [file pone.0130249.s001.tif]
